# All the Way: A Decade of SIRT1 in Breast Cancer

**DOI:** 10.3390/biomedicines14030671

**Published:** 2026-03-15

**Authors:** Giovanni Pratelli, Mauro Montalbano, Federica Affranchi, Chiara Occhipinti, Marianna Lauricella, Daniela Carlisi, Anna De Blasio

**Affiliations:** 1 Department of Biomedicine, Neurosciences and Advanced Diagnostics (BIND), Institute of Biochemistry, University of Palermo, 90127 Palermo, Italy; marianna.lauricella@unipa.it (M.L.); daniela.carlisi@unipa.it (D.C.); 2 Mitchell Center for Neurodegenerative Diseases, Departments of Neurology, Neuroscience and Cell Biology, University of Texas Medical Branch, Galveston, TX 77555, USA; mamontal@utmb.edu; 3 Laboratory of Biochemistry, Department of Biological, Chemical and Pharmaceutical Sciences and Technologies (STEBICEF), University of Palermo, 90127 Palermo, Italy; federica.affranchi@unipa.it (F.A.); chiara.occhipinti@unipa.it (C.O.)

**Keywords:** SIRT1, breast cancer, triple-negative breast cancer, SIRT1 modulators, miRNAs

## Abstract

Breast cancer (BC) is a highly heterogeneous genetic disease, comprising several subtypes with distinct features that significantly influence prognosis and treatment outcomes. Among these subtypes, triple-negative breast cancer (TNBC) is particularly aggressive and makes it resistant to many standard therapies. Epigenetic mechanisms, including acetylation and deacetylation, are crucial in regulating gene expression and maintaining normal cellular functions and are closely associated with BC progression. In this context, the histone deacetylases sirtuins (SIRT1-7) regulate key biological processes like genomic stability, inflammation, cellular senescence, and metabolic functions, increasingly linked to cancer. In particular, SIRT1 shows dual roles, functioning both as a tumor suppressor or an oncogene, contributing to cancer initiation, progression, and metastasis as well as chemotherapy resistance. Despite extensive research in the past decade, the exact role of SIRT1 in BC, especially in TNBC, remains controversial. Recent findings suggest that SIRT1 can be modulated not only through pharmacological approaches but also using natural extracts, offering potential alternative or complementary therapeutic strategies. Additionally, SIRT1 activity is regulated by a complex network of miRNAs, highlighting the need for further investigation. This review aims to summarize recent studies to identify key insights into the role of SIRT1 and explore it as a potential therapeutic target in BC.

## 1. Introduction

Human breast cancer (BC) is a genetic disease, characterized by vast molecular heterogeneity and represents, today, the most common and frequent type of cancer diagnosed amongst females worldwide. It also remains a prevailing cause of cancer mortality in women followed by lung and colorectal cancer [1]. The occurrence of BC is usually associated with multiple hereditary and environmental etiologic factors including hormonal disorders, genetic mutations, unhealthy eating habits and precancerous lesions [2,3]. Several techniques used in the field of molecular biology led to the identification of inherited gene mutations and to the establishment of genetic markers such as *BRCA1*, *BRCA2*, *DBC-1*, *TP53*, *PTEN*, *ATM*, *CHEK2*, and *HRAS1*, which could predispose a patient to the disease [4,5]. Moreover, BC is also a complicated genetic process regulated at various development stages [6]. BC comprises several distinct subtypes that have different genetic, morphological, and pathological characteristics with different consequences for the prognosis and lethality of the disease [7]. Currently, supported by the use of microarray techniques that allow the discrimination of BC subtypes based on the expression of key molecular markers, many trials use a panel of antibodies that include ER, PR, HER2/neu, cytokeratin 5/6, EGFR, and Ki-67 to assign breast tumors to the various molecular subtypes (Table 1), which are classified in an ascending order of aggressiveness: luminal A; luminal B (HER2−); luminal B (HER2+); HER2−enriched; and basal-like (triple-negative). Thus, since BC is characterized by complex molecular heterogeneity [8,9], therapeutic approaches are based on clinical and pathological features including a combination of surgery with radiotherapy, endocrine therapy, and/or chemotherapy [10].

Alterations in epigenetic mechanisms such as methylation, demethylation, acetylation and deacetylation of chromatin, histones, and non-histone proteins play a crucial role in gene expression regulation to determine normal cell physiology and cell type identities and to modulate responsiveness to internal and external stimuli [11]. However, numerous studies show a broad role of epigenetic modifications in tumor initiation, progression, and metastasis [12,13,14,15], and how these epigenetic alterations may contribute to hallmarks that must be acquired for the development of a type of human cancer [16]. For example, aberrant alterations in reversible histone acetylation and deacetylation have been found in BC; these alterations are associated with early and/or late BC cell phenotypes, estrogen response and epithelial–mesenchymal transition (EMT), but also are related to the expression of some genes involved in cell growth, cell cycle, DNA repair, apoptosis and metastasis [17,18,19]. Recently, studies of epigenetic modifications and their implications in BC growth and metastasis have motivated research on the development of cancer drugs possessing epigenetic modulatory activities [20].

The histone acetylation and deacetylation modifications are controlled by a balance of enzymatic activity between histone acetyltransferase (HATs) and histone deacetylase (HDACs) of both histone and non-histone proteins. For this reason, HDACs are also referred to as lysine deacetylases (KDACs), emphasizing their enzymatic function rather than their substrates, which include non-histone proteins, as is also the case for sirtuins [21]. The HDAC family includes three classes of proteins, i.e., classes I, II, and IV [22], while class III proteins are called sirtuins (SIRT1-7) and are located between the nucleus, mitochondria, and cytoplasm.

Sirtuins (SIRTs) are nicotinamide adenine dinucleotide (NAD^+^)-dependent class III HDACs or KDACs that belong to the silent information regulator 2 (Sir2) family; Sir2-like proteins are phylogenetically conserved in eukaryotes, prokaryotes, and archaea [23]; moreover, they possess a conserved domain for core catalysis with several sequence motifs, and for this feature, SIRTs are divided into four classes phylogenetically [24]. Seven Sir2-like proteins (SIRT1-7) have been identified in mammalian cells and are distinguishable both structurally and based on their different subcellular localization. SIRTs contain two enzymatic activities: mono-ADP-ribosyltransferase and histone deacetylase; moreover, they contain a small zinc-binding domain and a large Rossmann fold domain, which together form a specific structure to unite NAD^+^ as a cofactor. The sirtuin-mediated deacetylation reaction combines lysine deacetylation with NAD^+^ hydrolysis: by transferring the acetyl group to the ADP-ribose and NAD^+^ hydrolysis yields the deacetylated substrate, 2′-O-acetyl-ADP-ribose and nicotinamide (NAM), which inhibits sirtuin activity [25]. SIRTs play a central role in several biological processes such as cell survival, apoptosis, the regulation of DNA repair, genomic stability, inflammation, cellular senescence, metabolic regulation, stress response and cancer [26,27,28,29,30]. In recent years, the interest toward SIRTs is expanded thanks to outcomes obtained that have highlighted their roles in regulating physiological and pathological conditions. A member of the sirtuin family, SIRT1, is the one with the greatest relevance [31].

This review integrates and analyzes research from the past decade to provide a comprehensive overview of the involvement of SIRT1 in BC. Particular emphasis is placed on describing the recently identified mechanisms through which SIRT1 may modulate tumor progression, as well as examining its potential as a therapeutic target.

## 2. SIRT1 in Human Diseases: An Overview

SIRT1 is the most studied among the SIRTs. SIRT1 removes the acetyl group from the ε-amino group of lysine residues in both histone and non-histone proteins as targets and, although its primary role is as a nuclear deacetylase, produces two nuclear localization-export signals that allow it to shuttle between the nucleus and cytoplasm [32]. SIRT1 is largely recognized as a critical epigenetic regulator of gene expression and protein activities in several biological processes involved in metabolic disorders, DNA damage, neurodevelopment and brain senescence, cellular stress, aging and tumorigenesis [33]. The main histone and non-histone substrates and the biological findings of SIRT1 proteins are summarized in Table 2.

SIRT1 is involved in numerous neuronal processes and plays a role in several neurological disorders, including Alzheimer’s disease, Huntington’s disease, and Parkinson’s disease. In this context, SIRT1 exerts a marked neuroprotective action, as demonstrated in the mouse models of HD, where it modulates cellular survival, neuropathology, and the expression of brain-derived neurotrophic factors [34,35,36,37,38].

SIRT1 plays a critical role in aging and cellular senescence. Aging involves progressive structural and functional decline, including immune impairment, chronic inflammation, and circadian rhythm disruption [39]. SIRT1 delays cellular senescence and interacts with conserved longevity pathways, including AMPK, IGF-1 signaling, mTOR, and FOXO transcription factors [40,41].

Aging is also linked to obesity, oxidative stress, chronic inflammation, and metabolic dysregulation. It was reported that nutritional status modulates SIRT1; insulin and ChREBP suppress its expression, whereas fasting and glucagon signaling induce transcription via CREB/CRTC2. Genetically modified mice demonstrate SIRT1’s crucial role in metabolic homeostasis [42,43].

Type 2 diabetes mellitus (T2DM) represents a major metabolic disorder affecting a significant proportion of the adult population worldwide. It was recently demonstrated that, in animal models, a selective activator of SIRT1, SRT2104, improves glycemic control in adults with T2DM and increases insulin sensitivity [44,45]. Recent studies in mouse models of T2DM have demonstrated that SIRT1 enhances mitochondrial function and biogenesis, thereby conferring protection against diet-induced obesity and insulin resistance.

SIRT1 has also been shown to directly bind to and deacetylate autophagy-related proteins, including ATG5, ATG7, and ATG8, thereby promoting mitophagy and facilitating the selective removal of dysfunctional mitochondria. In addition, SIRT1 stimulates the autophagic pathway through the deacetylation of the transcription factors FOXO1 and FOXO3a. Furthermore, substantial evidence supports a protective role for SIRT1 in the cellular response to oxidative stress, promoting mitochondrial biogenesis [46].

Moreover, SIRT1 has been shown to play an important role in adipose tissue, especially in brown adipose tissue, which exhibits high mitochondrial SIRT1 expression, modulating the key regulators of mitochondrial biogenesis, such as PGC-1α and PPAR-γ, and promoting the trans-differentiation (browning) process [47].

SIRT1 has emerged as a key regulator of the DNA damage response, functioning both as a histone deacetylase at sites of DNA damage and as a deacetylase of non-histone proteins directly involved in DNA repair pathways. SIRT1-mediated histone deacetylation promotes alterations in chromatin compaction, whereas the deacetylation of non-histone substrates modulates protein activity and stability. Through these complementary mechanisms, SIRT1 contributes to the protection against DNA damage and to the efficient cellular response to genotoxic stress [48].

It is now well established that SIRT1 plays a crucial role in cancer, acting as a context-dependent modulator with dual and often opposing functions, either as a tumor promoter or as a tumor suppressor. These contrasting roles depend on the cancer type, the targeted signaling pathways, and the specific cellular context. The oncogenic contribution of SIRT1 to tumor growth and progression is largely attributable to its ability to repress tumor suppressor proteins, such as p53 and p73, and to modulate key signaling pathways including Wnt, TGF-β, and NF-κB. Conversely, SIRT1 has also been shown to inhibit oncogenic genes and proteins, thereby revealing its tumor-suppressive potential as a histone/protein deacetylase. Within this dual framework, SIRT1 regulates fundamental processes such as cell proliferation and tumor growth, as well as more advanced events including cell migration, invasion, metastasis, and drug resistance [49,50].

Collectively, SIRT1 acts as a central regulator at the intersection of neurodegeneration, aging, and metabolism, highlighting its potential as a therapeutic target for multiple age-related disorders.

## 3. Dual Role of SIRT1 in Breast Cancer

Described as the “regulator of regulators”, SIRT1 is markedly involved in various cellular processes such as gene regulation and genome maintenance, cell differentiation, DNA repair, metabolism, aging and cancer development. Due to its deacetylase activity, dysfunctional SIRT1 can suppress or promote initiation, progression and metastasis in different human cancer types including breast. Although several studies in the last decade highlight SIRT1’s implication in human BC, its role remains very controversial and yet unclear. However, the SIRT1 downstream substrates or upstream regulators as well as its cellular distribution and the tumor types seem to contribute to the SIRT1 context-specific role in BC [51].

**Figure 1:**
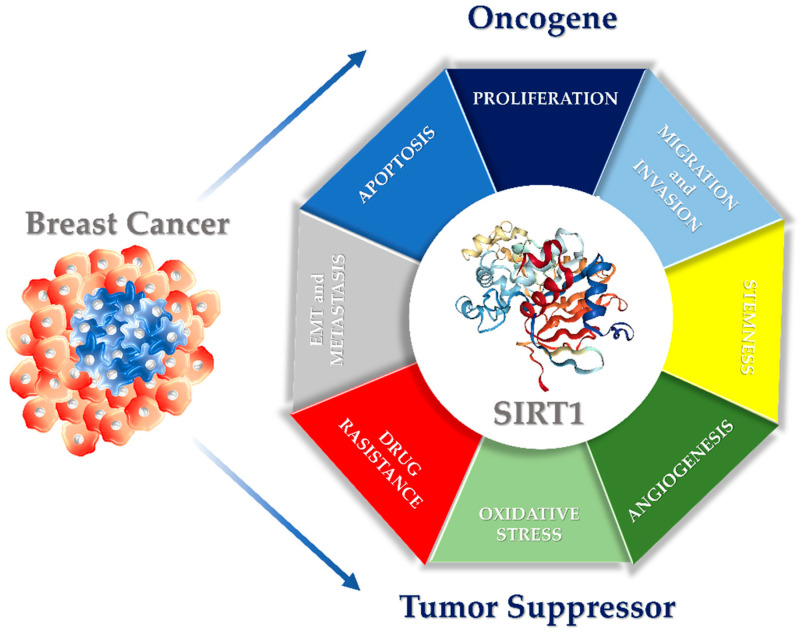
Conceptual wheel model illustrating the dual role of SIRT1 in BC outcomes. As the driver of the wheel, SIRT1 can direct the system toward oncogenic signaling (forward rotation) or tumor-suppressive activity (backward rotation), depending on the tumor context.

However, within the framework of the other reported findings, SIRT1 emerges as a versatile regulator rather than a molecule with a fixed oncogenic or tumor-suppressive identity. Its involvement in pathways controlling tumorigenic behavior and stemness in BC suggests a regulatory function that adapts to the tumor phenotype (luminal, HER-2 positive, or TNBC) and the molecular context of the cell (Figure 1). In particular, the cellular p53 status appears to shape SIRT1 activity and downstream signaling, directing distinct transcriptional programs that influence proliferation, survival, and differentiation. As a result, SIRT1 may support growth-inhibitory and pro-apoptotic responses in certain settings while driving alternative adaptive mechanisms in others [52].

### 3.1. Oncogenic SIRT1

While SIRT1 has long been regarded as a guardian of genome integrity and metabolic balance, recent discoveries suggest that its role is far more multifaceted than once believed. Its apparent dualism seems to arise from the surrounding molecular environment and from the crosstalk between metabolic stress, hormonal pathways, and epigenetic mechanisms. These observations depict SIRT1 not as a passive stabilizer but as an active driver of oncogenic transformation and BC progression processes.

Shi et al. identify an SIRT1-PRRX1-KLF4-ALDH1 circuit that links cancer stem cells (CSCs) with chemoresistance, metastasis, and aging. SIRT1 deacetylates and stabilizes PRRX1, an inducer of EMT, which subsequently suppresses the transcription of the core stemness factor KLF4. In particular, the loss of SIRT1 function leads to the destabilization of PRRX1, the release of KLF4 from inhibition, and the activation of ALDH1, which is associated with stemness, drug resistance and metastasis. In this context, while KLF4 levels are inversely correlated with SIRT1, PRRX1 levels are positively correlated, suggesting an SIRT1-centered circuit that regulates stemness in BC [53].

Based on the promotion of EMT by SIRT1, Choupani et al. reveal that oleuropein (OLEU), a compound known for its anticancer and protective effects, influences cell migration by downregulating SIRT1. This reduction in SIRT1 expression plays a significant role in suppressing EMT, leading to a decrease in the migratory capacity of MCF-7 breast cancer cells. The findings indicate that SIRT1 regulates the movement of BC cells, and that its suppression by OLEU effectively hinders metastasis. Moreover, this study proposes the SIRT1 potential to enhance treatment efficacy, especially when used in conjunction with chemotherapy drugs like doxorubicin (DOX) [54].

Beyond stemness regulation, SIRT1 is involved in modulating estrogen signaling through its interaction with estrogen receptor alpha (ERα), which deacetylates to influence downstream gene expression. The ERα transcriptional regulation of SIRT1 also appears to involve estrogen-related receptors (ERRs), especially ERRβ, which shares significant DNA-binding domain homology with ERα, activating the regulatory elements. In models where ERRβ is overexpressed, SIRT1 transcription and activity are significantly enhanced, an effect further amplified by the presence of the coactivator PCAF. This upregulation correlates with the increased rates of cellular proliferation, motility, and colony formation, which are decreased by the presence of SIRT1 inhibitors supporting oncogenic processes and indicating a functional role in driving tumor aggressiveness [55].

SIRT1 plays a critical role in maintaining the survival and metastatic adaptability of tumor cells, especially in adapted suspension cells (ASCs). Park et al. demonstrated that the elevated expression of SIRT1 safeguards ASCs from apoptotic cell death by inhibiting NF-κB activity, which normally supports survival pathways, and by lowering the levels of ROS, thereby reducing oxidative damage. Notably, SIRT1 levels are significantly higher in ASCs cultured over extended periods compared to those in short-term cultures, underscoring its importance in metastatic adaptation. Consequently, the overexpression of SIRT1 aids in cellular survival and boosts the metastatic capability of tumor cells, making it a crucial player in their resistance to apoptosis [56].

Higher SIRT1 levels in tumors correlate with poor prognosis. SIRT1 regulates FOXO3 acetylation, a key tumor suppressor playing an important role in lapatinib resistance. Mahmud et al. observed that, when SIRT1 deacetylates FOXO3, its activity is reduced, impairing the response to lapatinib in HER2−positive BC. In sensitive cells, inhibiting SIRT1 or silencing it with siRNAs increases FOXO3 acetylation, enhancing drug efficacy. In contrast, EP300 overexpression, which promotes FOXO3 acetylation, boosts drug susceptibility in sensitive cells but not in resistant ones [57]. Similarly, Dilmac et al. identified that SIRT1 expression was significantly elevated in both primary tumors and metastatic tissues, particularly in the 67NR metastatic cell line, while tumor suppressors like p21 and p53 were reduced in metastatic sites, suggesting that SIRT1 may promote an oncogenic shift during metastasis. The study also highlights the interaction between SIRT1 and FOXO proteins, which regulate pathways involved in cell cycle, proliferation, and invasion. SIRT1 appears to enhance the expression of E2F1, supporting tumor cell survival and migration, thus driving aggressive BC behavior. Bioinformatics analysis further supports SIRT1 involvement in metastatic pathways, indicating its critical role in the molecular cascades that promote tumor spread. Elevated SIRT1 expression in metastatic tissues underscores its oncogenic potential and suggests that it plays a crucial role in driving metastasis in advanced cancer stages [58].

SIRT1 is also a key regulator of aromatase expression in breast adipose fibroblasts within ER+ BC. Kaiser et al. demonstrated that, unlike PARP-1, which activates the I.3/II promoter via parylation under low-NAD^+^ conditions, SIRT1 independently enhances promoter activity through NAD^+^-dependent deacetylation. Although both enzymes compete for promoter binding, SIRT1 acts through a distinct, non-redundant mechanism, functioning as a metabolic sensor of redox state. Their inhibition alters the NAD^+^/NADH ratio, highlighting a regulatory balance where SIRT1 counteracts PARP-1 to control local estrogen synthesis [59].

In ER+ BC cells such as MCF-7, elevated SIRT1 activity has been associated with increased resistance to cell death. In an effort to explore therapeutic strategies targeting this pathway, Kojja et al. have focused on novel 2-(diarylalkyl)aminobenzothiazole derivatives, which have shown promising anticancer potential in MCF-7 cells, revealing a high binding affinity for SIRT1. The compounds reduced SIRT1 protein levels while simultaneously enhancing p53 acetylation, thereby reactivating its tumor suppressor functions, increasing apoptosis and autophagic cell death [60].

By analyzing the interaction and correlation between SIRT1 and SRC on human BC prognosis, Tan et al. studied their roles in cell proliferation, tumor invasion, and metastasis. They found that the overexpression of both SIRT1 and SRC is correlated with poor prognosis in human BC, especially in luminal type. Combined SIRT1/SRC inhibition significantly suppressed cell migration and invasion in MCF-7 cells with a positive association between SIRT1 and SRC both in BC tissues and in MCF-7 cells [61].

Some studies classify SIRT1 as a tumor suppressor, while others suggest it may act as a proto-oncogene. Elevated SIRT1 expression has been strongly linked to the development of resistance to tamoxifen (TAM) in BC cells, with high SIRT1 levels correlating with poorer clinical outcomes in patients undergoing TAM therapy. A study by Zhou et al. confirmed a positive correlation between SIRT1 and SRC expression and the functional interaction between these two factors in driving resistance mechanisms. While overexpression of SRC was found to intensify TAM resistance, this effect could be mitigated by SIRT1 inhibition, highlighting the latter central role in the resistance pathway. Both in vitro and in vivo experiments confirmed that downregulating either SRC or SIRT1 can reverse resistance to TAM and effectively suppress tumor cell proliferation [62].

Xu et al. have investigated the significant role of SIRT1 in BC by exploring the effect of SIRT1 on *POLD1*, the gene coding for DNA polymerase d catalytic subunit p125. Immunohistochemical analyses provided the evidence that the expression level of SIRT1 protein is higher in BC tissues than in normal tissues. However, the overexpression of SIRT1 enhanced the aggressiveness of the human BC cell line MCF-7; the knockdown of SIRT1 using shRNA reduced the abovementioned cellular processes. In this context, results revealed a positive correlation between SIRT1 expression and p125. Indeed, the overexpression of SIRT1 decreased the p53 level while increasing the expression level of POLD1/p125; conversely, the silencing of SIRT1 produced the opposite effect, suggesting an oncogenic function of SIRT1 in BC cells [63].

Emerging evidence suggests that SIRT1 plays an active role in BC cell responses to external stimuli, particularly those involving adrenergic signaling and receptor regulation. Liu et al. demonstrated in BC cell lines such as MDA-MB-453 and SKOV3 that exposure to epinephrine leads to a marked increase in the expression of SIRT1. This upregulation is closely linked to a decrease in miR-199a-5p, a microRNA that normally suppresses SIRT1 levels. The stimulation of β2-adrenergic receptors activates the PI3K/AKT pathway, which contributes to the reduction in miR-199a-5p and consequently elevates SIRT1 expression. Elevated SIRT1 then acts to enhance the transcription of ADAM10, a metalloprotease responsible for cleaving the ectodomain of the HER2 receptor. This sequence of events suggests that SIRT1 plays a crucial role in regulating HER2 shedding in BC cells, potentially affecting downstream signaling pathways that influence tumor behavior in response to adrenergic signals [64].

Jin et al. identified SIRT1 as a key contributor to DOX resistance in BC. In resistant MCF-7/ADR cells, SIRT1 expression is significantly elevated compared to sensitive counterparts, suggesting a role in promoting drug tolerance. Functional studies demonstrate that silencing SIRT1 restores sensitivity to DOX, both in vitro and in vivo, while its overexpression sustains resistance, highlighting its influence on treatment outcomes. SIRT1 enhances the activation of the AKT signaling pathway, increasing its phosphorylation and contributing to the maintenance of the resistant phenotype. The pharmacological inhibition of AKT partially reverses SIRT1-mediated resistance, confirming the functional relevance of the SIRT1-AKT axis and underscoring the potential of SIRT1 as a predictive biomarker in BC to overcome chemoresistance and improve the efficacy of DOX-based therapies [65]. In addition, SIRT1 activity suppresses the DOX-associated rise in senescence markers (p53, p21, and SA-β-Gal), indicating that SIRT1 restrains the establishment of a senescent state. This effect is tightly linked to the restoration of autophagic flux, which DOX normally impairs. SIRT1 enhances p62 accumulation and normalizes the LC3II/LC3I ratio. At the signaling level, SIRT1 counteracted the DOX-induced activation of PI3K/AKT/mTOR signaling pathway, leading to enhanced autophagic activity. Pharmacological modulation further supports this mechanism: PI3K inhibition reinforces, whereas AKT activation diminishes, promoting the SIRT1-mediated suppression of senescence in MCF-7 cells [66].

Moreover, SIRT1 regulates the autophagy pathway mediated by TFEB in BC. A retrospective study shows a positive association between SIRT1 with both TFEB and CARM1. Tumors with very low SIRT1 levels exhibit correspondingly reduced TFEB and CARM1 expression, which correlates with improved survival in patients receiving chemotherapy suggesting that elevated SIRT1 may promote chemoresistance by enhancing autophagy-driven survival mechanisms in BC cells. In addition, the expression of SIRT1 also differs according to the molecular subtype: basal-like and HER2−enriched tumors display lower levels of SIRT1 expression compared to luminal tumors, reflecting potential differences in autophagic activity and response to treatment. The observed positive correlation between SIRT1 and Beclin-1 further supports its role in autophagy initiation, which may enable tumor cells to withstand chemotherapeutic stress [67]. SIRT1 also coordinates the balance between autophagy and inflammation in the tumor microenvironment. In a recent study, Das et al. found that, in Ehrlich’s Ascites Carcinoma (EAC) cells, melatonin-induced ROS activated SIRT1, which interacts with NF-κB, a key pathway for regulating both processes. This interaction promotes tumor progression by modulating inflammatory responses and autophagic activity. Melatonin disrupts the NF-κB/SIRT1 interaction, inhibiting the IL-6/STAT3/NF-κB pathway and reversing the pro-inflammatory environment. This shift enhances autophagic responses, contributing to a more balanced regulation of autophagy, inflammation, and EMT. Thus, SIRT1 acts as a key modulator of tumor progression, apoptosis, and autophagy in EAC [68].

Wang et al. demonstrate that elevated NNMT expression enhances both the abundance and enzymatic activity of SIRT1, allowing the BC cells to better withstand the cytotoxic effects of chemotherapeutic drugs such as DOX and paclitaxel (PTX). This increase in SIRT1 activity supports cell survival by limiting apoptosis and sustaining the capacity for colony formation in BC cell lines. Importantly, the disruption of SIRT1 function either through the selective inhibitor or siRNA-mediated knockdown reverses NNMT-induced chemoresistance [69].

SIRT1 also plays a crucial role in BC progression through its activation by the CD44 receptor. When CD44 binds its ligand hyaluronan, it induces SIRT1 expression, thus promoting cell proliferation, migration, and metastasis. Studies using a tetracycline-inducible CD44 system in mouse models have shown that CD44 activation upregulates SIRT1, which then modulates key oncogenic pathways such as PI3K/AKT, MAPK/ERK, and Wnt signaling. These pathways drive tumor growth, cell cycle progression, and invasion promoting the expression of oncogenes like Cyclin D1 and C-Myc, which support metastasis [70].

Although SIRT1 have been widely studied, its prognostic value in BC remains controversial. However, emerging evidence suggests that elevated SIRT1 expression levels are closely associated with unfavorable clinical outcomes. A comprehensive meta-analysis involving over 6000 patients from 22 studies revealed that high SIRT1 expression correlates with significantly reduced overall and disease-free survival. These findings were consistent across both univariate and multivariate analyses, highlighting SIRT’s potential role in tumor progression. Subgroup analysis further confirmed its link to poorer prognosis, suggesting that SIRT1 may contribute to more aggressive disease behavior. As such, SIRT1 may serve not only as a biomarker for risk stratification but also as a potential target for improving personalized treatment approaches in BC [71].

Collectively, these findings provide strong evidence that SIRT1 acts as a multifaceted oncogenic driver in BC.

### 3.2. Tumor Suppressor SIRT1

SIRT1 has emerged as a pivotal modulator of BC biology, exerting multifaceted effects on cellular proliferation, apoptosis, and therapeutic response. Increasing evidence points to a tumor-suppressive dimension of SIRT1 activity, suggesting that its proper regulation may counteract key oncogenic processes. Through its interactions with crucial metabolic and signaling pathways, SIRT1 contributes to the maintenance of genomic stability, regulation of cell cycle checkpoints, and enhancement of DNA repair, positioning it as a molecular safeguard against tumor progression. Nonetheless, its function is highly context-dependent, shaped by cellular microenvironment, hormonal signaling, and redox state.

In addition to its metabolic regulatory functions, SIRT1 has also been shown to influence hormone receptor signaling in BC and it is required for the growth of ERα+ BC. The SIRT1-mediated deacetylation of ERα results in a repressive effect on its transactivation, which has a critical role in the progression of BC. Xu et al. demonstrated that checkpoint suppressor 1 (CHES1) interacting with ERα represses its transactivation by forming a complex with ERα and SIRT1. Their results further indicate that CHES1 facilitates the recruitment of SIRT1 and promotes the SIRT1-mediated deacetylation of ERα in ERα+ MCF-7 and T47D cells [72].

Furthermore, SIRT1 activity has been connected to obesity-related mechanisms influencing ER signaling and aromatase expression. In the breast tissue of obese women, the levels of aromatase as well as those of prostaglandin E2 and hypoxia-inducible factor 1α (HIF-1α) are increased, which contribute to the induction of aromatase in adipose stromal cells (ASCs). Subbaramaiah et al. showed that SIRT1 plays a role in the regulation of aromatase expression by binding, deacetylating, and thereby inactivating HIF-1α. They demonstrated, in ASCs, that treatment with prostaglandin E2 reduced SIRT1 levels, which are linked to the increased levels of acetyl-HIF-1α as well as enhanced aromatase gene transcription [73].

Beyond its influence on hormonal regulation, SIRT1 also exerts epigenetic control in BC, particularly through histone deacetylation. It is known that the complexity of carcinogenesis involves profound epigenetic deregulations that contribute to the tumorigenesis process, including the deregulated acetylation of histones H3 and H4. In a study, Rifaï et al. elucidated SIRT1’s epigenetic role by evaluating the epigenetic marks of histones H3 and H4 in five molecular subtypes of BC and a set of 135 human breast tumors. They observed that SIRT1 modulates and regulates its H3 acetylated targets in a subtype-specific manner with an inverse correlation with three epigenetic marks: H3k4ac, H3k9ac and H4k16ac expression patterns. Moreover, SIRT1 knockdown increased the histone acetylation levels at six BC-related gene promoters including BRCA1 [74].

This relationship between SIRT1 and BRCA1 extends beyond epigenetic regulation, as demonstrated by its direct enzymatic interaction with BRCA1. As shown by Lahusen et al., SIRT1 deacetylates the acetylated form of BRCA1, suggesting that it is a specific deacetylase of BRCA1. Additionally, the same authors observed that SIRT1 plays a role in BRCA1-associated tumorigenesis, and, in a mouse model, the loss of SIRT1 resulted in DNA damage and tumorigenesis [75].

Interestingly, the connection between SIRT1 and BRCA1 appears reciprocal, with BRCA1 also modulating SIRT1 transcription. BRCA1 positively regulates the SIRT1 transcription in a tissue-specific manner. Considering this, SIRT1 is not only an anti-apoptotic protein survivin inhibitor but also its expression is necessary for telomer elongation. BRCA1 haplodeficient human breast epithelial cells showed significant reduction in telomer length as well as chromosomal aberrations. In addition, reduced SIRT1 expression upregulates surviving, favoring tumorigenic anti-apoptotic features [76].

SIRT1 function in BC may depend on other cellular factors, such as CD36 expression levels. Yao et al. showed how the contrary effects of SIRT1 could be dependent on the CD36 expression level in the MCF-7 cell line. The authors demonstrated that SIRT1 was upregulated while CD36 decreased in BC tissues, compared with normal adjacent tissues. In this study, the treatment with RES, an SIRT1 activator, increased the proliferation of MCF-7 cells as well as the concentration of CD36 while dexmedetomidine treatment increased cell proliferation but downregulated the expression of SIRT1/CD36. However, upregulation of SIRT1 via RES pretreatment could suppress cell proliferation stimulated by dexmedetomidine accompanied with CD36 upregulation in MCF-7 cells, suggesting that SIRT1 overexpression was capable of inhibiting cell proliferation [77].

SIRT1 is also involved in the regulation of circadian rhythm and drug resistance mechanisms. Xiang et al. showed that SIRT1 melatonin-mediated induction as well as STAT3 deacetylation at K685 by SIRT1 in MCF-7 breast tumor xenografts describe an effective mechanism by which melatonin inhibits STAT3-mediated PTX resistance in BC and how dim light in night-mediated circadian melatonin disruption by DNA methylation can promote drug resistance in BC [78]. Other studies have confirmed the link between SIRT1, STAT3, and therapeutic response in BC. Wang et al. determined the SIRT1 expression levels in BC tissues and cells with cystathionine γ-lyase (CSE) expression, which is highly expressed in BC, promoting tumor development and progression. The authors showed that CSE expression was negatively associated with SIRT1 in BC. Their results revealed that I157172, a CSE inhibitor, significantly inhibited the growth, proliferation, and migration in MCF-7 cells by SIRT1 upregulation and consequent the deacetylation and inactivation of STAT3 [79].

SIRT1 modulation in apoptotic pathways has also been evaluated, particularly in the context of hormone therapy. Conversely, in research conducted by Ghatreh Samani et al., TAM was shown to upregulate SIRT1 expression, an effect further enhanced by Lauryl Gallate (LG). These findings were validated through SIRT1 inhibition. Interestingly, while SIRT1 inhibition had only a modest effect on apoptosis levels, the combined treatment with TAM and LG significantly influenced BCL-2 expression more than SIRT1 inhibition alone. This suggests that the pro-apoptotic effect of TAM and LG may be mediated, at least in part, by SIRT1 regulatory influence on BCL-2 [80].

Apoptosis and autophagy modulation by SIRT1 also emerge under nitrosative stress conditions. Investigating the role of autophagy under nitrosative stress, Chakraborty et al. observed induction of autophagy coupled with cell death in an SIRT1-AMPK-p53-dependent manner following NO donor treatment in MCF-7 cells. As a result of SIRT1 inhibition activity and SIRT1 knockdown, autophagy markers and phospho-AMPK were downregulated, and the accumulation of acetylated p53 increased cell viability, suggesting a regulatory complex between AMPK, SIRT1 and p53 in autophagy induced by nitrosative stress [81]. Moreover, in BC cells expressing wild-type p53, the regulatory role of SIRT1 extends beyond its known deacetylase activity; in particular, the C-terminal region of p53 includes several lysine residues whose acetylation influences the protein stability and interaction profile. Kim et al. found that, when SIRT1 is inhibited or silenced in MCF-7 cells, acetylation levels within this domain rise, leading to an accumulation of both p53 and its downstream effector p21. However, despite this increase in expression, the direct association between p53 and p21, a critical regulator of cellular function, is noticeably weakened. These observations point out a role for SIRT1 in modulating the p53-p21 interaction, by a specific acetylation pattern that supports this complex formation [82].

These findings also align with evidence linking SIRT1 downregulation to senescence processes. SIRT1 helps maintain cellular stability and prevents premature senescence, which is often associated with tumor suppression and aging. In a recent study, Li et al. showed that Neratinib, a tyrosine kinase inhibitor used to treat HER2−positive BC, induces cellular senescence in AU565 cells. This effect is linked to mitochondrial dysfunction, ROS increase, and ATP level decrease, which subsequently cause DNA damage and reduced telomerase activity. A significant observation is that Neratinib reduces SIRT1 levels, which seems to play a pivotal role in triggering senescence. The downregulation of SIRT1 leads to the acetylation of p53 and the activation of p21, both of which are key players in the senescence process. However, when SIRT1 is overexpressed in AU565 cells, the senescence-inducing effects of Neratinib are reversed, underscoring the critical role of SIRT1 in this cellular response [83].

Finally, SIRT1 function in BC is modulated not only at the gene expression level but also through post-translational modifications. Kang et al. suggest that, although SIRT1 mRNA levels tend to decrease in invasive tumors compared to normal tissue, its enzymatic function can be enhanced under stress conditions via ISGylation, a process involving the modifier ISG15, an interferon-stimulated ubiquitin-like protein. These studies in MCF-7 cells show that treatment with DNA-damaging agents like DOX induces ISG15 expression and promotes SIRT1 ISGylation. This modification increases SIRT1 deacetylase activity by reducing its interaction with DBC1, a negative regulator, enabling a more effective deacetylation of substrates such as p53. These results indicate that ISGylation acts as a switch to activate SIRT1 during chemotherapy, potentially influencing tumor cell behavior and response to treatment in BC [84].

Overall, current evidence portrays SIRT1 as a pivotal guardian of cellular equilibrium and a key suppressive force in breast tumor biology. Through its deacetylating activity, SIRT1 influences fundamental regulators such as p53, ERα, BRCA1, and STAT3 helping to restrain aberrant proliferation, sustain genomic stability, and modulate apoptotic and inflammatory dynamics. Although its behavior may appear context-dependent, the prevailing data suggest that preserving or restoring SIRT1 function favors antineoplastic outcomes.

## 4. Implications and Dual Role of SIRT1 in TNBC

The lack of specific therapeutic targets is a major challenge in improving outcomes for TNBC patients, and SIRT1 plays a key role in tumor invasion, metastasis, and prognosis, making it a promising prognostic factor and a potential therapeutic target for TNBC. However, SIRT1 has been reported to play a dual role in BC including TNBC, and its expression is significantly elevated in tissues and cell lines, with strong correlations to aggressive clinical parameters, such as a higher histological grade, a larger tumor size, and lymph node metastasis [85].

Across BC subtypes, SIRT1 was predominantly localized in the nucleus, with nuclear and cytoplasmic expression closely aligned in luminal A, luminal B, and TNBC. Elevated nuclear SIRT1 was associated with poorer disease-specific survival in HER2−enriched BC, while increased cytoplasmic expression in the same subtype correlated with a larger tumor size and lymph node involvement, indicating a role in enhancing local aggressiveness. Conversely, SIRT1 expression was more frequent in low-grade tumors and aligned with better prognosis in luminal A disease, suggesting that SIRT1 participates in distinct signaling pathways whose impact varies by tumor subtype, reinforcing its dual and context-specific role in BC progression [86].

Using its deacetylase function, SIRT1 regulates FOXO3a, a key tumor suppressor. Deacetylation by SIRT1 prevents FOXO3a degradation and supports its nuclear localization, enhancing its DNA-binding activity and promoting the transcription of genes involved in cell cycle arrest, apoptosis, and oxidative stress response. Beyond FOXO3a, SIRT1 also modulates other crucial proteins like p53 and Ku70, influencing DNA repair and cell survival. In TNBC, high SIRT1 expression correlates with more aggressive features, such as high histologic grade, possibly through FOXM1 regulation. Conversely, high nuclear expression of both FOXO3a and SIRT1 is linked to lower histologic grades and better prognosis in ER+ PR+ HER2− BC, suggesting that while SIRT1 may drive aggressive tumor traits in TNBC, it also plays a tumor-suppressive role in other subtypes by enhancing FOXO3a activity [87].

### 4.1. Oncogenic SIRT1 in TNBC

In TNBC, SIRT1 demonstrates a context-dependent influence on tumor behavior. Its expression is significantly lower in TNBC compared to ER+ PR+ HER2− BC. According to this, reduced SIRT1 expression in ESR+ PR+ HER2− tumors is associated with worse survival and serves as an independent prognostic marker. However, TNBC cases with higher SIRT1 expression tend to have poorer clinical outcomes, suggesting a potential pro-tumorigenic role [88].

A recent study demonstrates that higher expression of SIRT1 correlates with increased resistance to DOX, such as MDA-MB-231 cells, and accelerated tumor growth in vivo. Upon DOX treatment, SIRT1 upregulation facilitates the nuclear translocation of NRF2, which in turn activates the antioxidant response element, leading to elevated GSH levels. This disruption of redox homeostasis helps maintain the survival and aggressiveness of DOX-resistant cells. Inhibition of SIRT1 effectively reverses these effects, preventing NRF2 activation and reducing GSH levels, thereby restoring redox balance. Additionally, the depletion of either SIRT1 or GSH abrogates the proliferative and invasive behavior of DOX-resistant cells. These findings underscore the role of SIRT1 in modulating not only redox signaling but also overcoming DOX resistance in TNBC [89].

In addition, with functional analyses using SIRT1-overexpressing and SIRT1-knockdown models, Jin et al. revealed that SIRT1 promotes cell proliferation, colony formation, cell cycle progression, and migration, while simultaneously suppressing apoptosis, demonstrating its broad influence on tumor cell survival and expansion both in vitro and in vivo. At the molecular level, SIRT1 directly interacts with AKT, facilitating its activation and thereby amplifying downstream pro-survival and proliferative signaling pathways. Notably, the partial inhibition of AKT attenuates the growth-promoting effects of SIRT1, highlighting that the oncogenic function of SIRT1 is at least partly mediated through the SIRT1-AKT axis and suggesting that SIRT1 not only supports tumor growth but also contributes to the enhanced metastatic potential of BC cells [90].

Another important point is the role of SIRT1 not only for its metastatic potential but also as an EMT modulator. In a study, Jin et al. explore the role of SIRT1 and its interaction with EMT markers in predicting lymph node metastasis and clinical outcomes in TNBC, using tissue samples from 319 TNBC patients and three human TNBC cell lines. The cohort was analyzed based on tumor size, AJCC stage, and adjuvant chemotherapy use. A combination of four proteins was identified as a significant predictor of lymph node metastasis and correlated with decreased disease-free survival, particularly in patients who received chemotherapy or had early-stage cancer. In vitro experiments showed that silencing SIRT1 with siRNA reduced tumor invasion in all three TNBC cell lines, with changes observed in EMT marker expression [91].

Furthermore, particularly in TNBC with BRCA1 mutations, SIRT1 plays a pivotal role in the progression of the disease. A study conducted by Xu et al. reveals that SIRT1 levels are elevated in cells with wild-type BRCA1a or the BRCA1a I26A mutant, but not in those with disease-associated Ubc9-binding mutants. Ubc9, an enzyme involved in SUMO conjugation, inhibits the expression of SIRT1 in TNBC. When Ubc9 is silenced, SIRT1 levels are restored, suggesting that Ubc9 negatively regulates SIRT1 expression independently of typical BRCA1 DNA repair functions. This regulation of SIRT1 is linked to EMT, a process that drives metastasis to the lungs and pleural effusion in TNBC. The study indicates that SIRT1-mediated Ubc9 repression, along with VEGF activation, reduction in caveolin-1, and suppression of ERα, forms a molecular pathway that enhances the metastatic capacity of BC cells [92].

Conversely, in this scenario, El-Ashmawy et al. showed that elevated SIRT1 activity supports a mesenchymal and invasive cellular phenotype, characterized by enhanced proliferation, motility, and angiogenic potential. Indeed, the inhibition of SIRT1 through sirtinol disrupts these malignant features, leading to a pronounced attenuation of the EMT. This is evidenced by reduced vimentin levels alongside the restoration of E-cadherin expression, indicating a shift toward a more epithelial and less aggressive cellular state. Moreover, SIRT1 blockade diminishes VEGF production and reduces phosphorylated AKT, suggesting that SIRT1 contributes to the maintenance of pro-survival and pro-migration signaling via AKT. The combined administration of montelukast and sirtinol yields a stronger antitumor response than either treatment alone, reinforcing the concept that SIRT1 sustains EMT dynamics, AKT pathway activation, and the overall oncogenic behavior of TNBC cells [93].

Moreover, epithelial–mesenchymal plasticity is crucial for metastatic progression of TNBC. Guo et al. identified an oxidative stress-responsive CBP/SIRT1 axis that regulates this process and metastasis under oxidative stress. CBP forms a dynamic complex with SIRT1 enabling the precise acetylation of ZEB1 at K1108. This modification promotes ZEB1 dissociation from the corepressor CtBP and its recruitment of CBP, converting ZEB1 into a transcriptional activator of epithelial genes. The resulting hybrid epithelial–mesenchymal phenotype supports metastasis by maintaining stromal interactions through partial EMT and enhancing redox balance to resist ferroptosis in TNBC [94].

In addition, SIRT1 modulates the post-translational regulation of several proteins including DVL proteins, central components of the Wnt signaling cascade, highly expressed in TNBC cell lines. Earlier observations indicate that SIRT1 influences both the abundance and functional behavior of DVL, implying that it may serve as a direct target for SIRT1-dependent lysine deacetylation. By adjusting the acetylation status of DVL, SIRT1 is positioned to shape its intracellular distribution and regulatory activity in tumor biology [95].

In TNBC cell lines such as MDA-MB-231, BT549, and MDA-MB-468, SIRT1 appears to function as a facilitator of tumorigenic behavior, sustaining cellular programs linked to stemness and metabolic adaptability. A study conducted by Du et al. reveals a clear antagonism between SIRT4 and SIRT1: when SIRT4 is active, it dampens glutamine-dependent metabolic pathways, resulting in a marked decrease in SIRT1 expression. Because SIRT1 influences chromatin dynamics and governs multiple gene networks involved in cellular fitness and metabolic regulation, its suppression is accompanied by reduced clonogenic growth, impaired sphere formation, and diminished invasive capacity. These observations indicate that SIRT1 contributes to the stem-like and aggressive phenotype characteristic of TNBC, whereas SIRT4 acts as a mitochondrial brake that restrains SIRT1-driven metabolic and epigenetic signaling [96].

SIRT1 emerges as a relevant regulatory node in TNBC stem-like cells. Visfatin-driven upregulation of SIRT1 in MDA-MB-231 cells enhances the self-renewal capacity and metastatic behavior of CSCs via the OCT4-SIRT1-p53 regulatory pathway. Indeed, visfatin treatment increased sphere-forming capacity and the upregulation of core stemness factors NANOG, SOX2, and OCT4 by the activation of SIRT1-SOX2 signaling axis. The suppression of these effects by the visfatin inhibitor FK866 supports SIRT1 functional involvement in visfatin-driven stemness and tumor progression [97].

Likewise, as shown by network pharmacology, docking analyses, and functional assays, Ginsenoside Rg3 (Rg3) interacts with proteins involved in breast CSC maintenance, including SIRT1. Computational docking suggests strong binding between Rg3 and SIRT1, implying that modulating SIRT1 may enhance Rg3 effects. Experimental data show that Rg3 reduces sphere formation, limits cell proliferation, and promotes apoptosis in MDA-MB-231 mammospheres. These effects correlate with a decrease in stemness markers like c-Myc, ALDH1A1, and NANOG, regulated partly by SIRT1. Furthermore, SIRT1 influences other key signaling pathways involved in breast CSCs, including the Hippo pathway highlighting SIRT1’s role in maintaining CSC identity [98].

Table 3 summarizes the factors that regulate or are regulated by SIRT1 in driving its oncogenic role in TNBC.

### 4.2. Tumor Suppressor SIRT1 in TNBC

SIRT1 plays an important regulatory role in lysosomal function and vesicle maturation, and its reduced expression contributes to a more invasive phenotype in TNBC. Latifkar et al. showed that the loss of SIRT1 impairs lysosomal acidification by lowering a critical V-ATPase subunit, leading to the inefficient degradation of multivesicular bodies (MVBs). As a result, enlarged MVBs preferentially fuse with the plasma membrane, releasing exosomes with altered cargo and hydrolases capable of remodeling the extracellular matrix. These changes explain how SIRT1 downregulation supports enhanced survival and invasion in aggressive BC cells [99]. In accordance with this, a phosphoproteomic analysis of BC-derived small extracellular vesicles revealed a higher enzymatic activity of SIRT1 in MDA-MB-231 in comparison to MCF-10A cells [100].

SIRT1 is involved in modulating RNA metabolism and lysosomal function. Specifically, it deacetylates the RNA-binding protein IGF2BP2, thereby stabilizing the RNA transcript of ATP6V1A, an essential subunit of the v-ATPase complex. This regulation is vital for maintaining proper lysosomal function, which is fundamental for cellular processes such as autophagy and protein degradation. However, the downregulation of SIRT1 levels leads to the hyperacetylation of IGF2BP2, promoting the recruitment of the exonuclease XRN2 and the subsequent degradation of ATP6V1A. This disruption of lysosomal function, coupled with the loss of SIRT1-mediated regulation, contributes to the increased aggressiveness of BC cells. Furthermore, the destabilization of RNA transcripts involved in endosomal trafficking and exosome production may further facilitate tumor cell invasion, suggesting that the role of SIRT1 in preserving RNA stability and lysosomal integrity is essential for regulating cell behavior [101].

SIRT1 also maintains redox balance and controls apoptosis. In models of DOX-induced cardiac injury, Hu et al. showed that the activation of SIRT1 by the myokine meteorin-like protein mitigates oxidative stress and prevents cardiomyocyte apoptosis through a cAMP/PKA-mediated mechanism. Crucially, this activation did not compromise the chemotherapeutic effects of DOX against 4T1 BC cells, either in vitro or in animal models. These observations indicate that SIRT1 can confer tissue-specific protection without diminishing the efficacy of anticancer treatment, reducing chemotherapy-associated toxicity without supporting tumor survival [102].

In the MDA-MB-231 cell line, SIRT1 functions as a central mediator linking mitochondrial disruption to adaptive signaling. Urra et al. observed that, when these cells encounter the mild OXPHOS uncoupling induced by a bromoalkyl ester of a hydroquinone derivative (FR58P1a), SIRT1 is required for AMPK activation, indicating its involvement in the metabolic response to mitochondrial stress. This SIRT1-AMPK axis supports a cytoprotective adjustment that helps the cells maintain viability under altered energetic conditions. At the same time, SIRT1-dependent AMPK activation reduces surface β1-integrin and consequently impairs fibronectin-driven adhesion and migration, an effect observed in MDA-MB-231 but not in non-tumoral MCF-10A cells. In this setting, SIRT1 integrates mitochondrial cues with downstream signaling to modulate both survival and motility, shaping the altered phenotype that emerges after sustained FR58P1a exposure [103].

In the SIRT1-AMPK axis scenario, SIRT1 is also identified as a regulator of the expression of the oncogenic protein metadherin (MTDH), in response to AMPK activation in TNBC cells. AMPK activation by AICAR or metformin inhibits MTDH expression via c-Myc. AMPK activates SIRT1 through the inhibition of Ser47 phosphorylation and activation of GSK3β, which, in turn, activates SIRT1. Activated SIRT1 deacetylates the tumor suppressor p53, which inhibits c-Myc, a transcriptional activator of MTDH. This cascade leads to a coordinated downregulation of MTDH. Inhibitor studies show that blocking AMPK, SIRT1, or GSK3β reverses this suppression, confirming their interconnected roles in regulating the c-Myc-MTDH axis [104].

Liang et al., in the treatment with Cyanidin-3-glucoside (C3G), a naturally occurring anthocyanin, have observed the upregulation of SIRT1, which subsequently suppresses NF-κB activity, a major driver of EMT. This SIRT1-dependent inhibition of NF-κB contributes to the reversal of EMT, as indicated by the increased expression of epithelial markers (E-cadherin, ZO-1), the decreased expression of mesenchymal markers (vimentin, N-cadherin), and the downregulation of EMT-related transcription factors (Snail1, Snail2). The experimental knockdown of SIRT1 abolishes both NF-κB suppression and EMT reversion, demonstrating that SIRT1 is essential for the anti-migratory and anti-invasive effects of C3G [105].

Another study underscores the pivotal role of SIRT1 in inhibiting metastasis and EMT in TNBC. The findings demonstrate that NAD^+^ supplementation activates SIRT1, resulting in reduced migration and cell invasion. This outcome is driven by the SIRT1-mediated deacetylation of p66Shc at K81, which leads to its inactivation and a decrease in the expression of mesenchymal markers, thereby impeding the EMT and reduced metastatic potential. Further investigation revealed that SIRT1 levels were notably lower in TNBC tissues, and the loss of SIRT1 was associated with more aggressive tumor behavior and poorer clinical outcomes while the overexpression of SIRT1 markedly reduced migration and invasion, supporting its role as a metastasis suppressor [106].

SIRT1 can be delineated as a pivotal regulatory factor, mediating the chemosensitizing effects observed following the inhibition of cystathionine-γ-lyase (CSE) in TNBC. The suppression of CSE activity by aurintricarboxylic acid (ATA) leads to the functional modulation of the SIRT1-associated signaling network, which in turn influences downstream STAT3 activity. Indeed, the attenuation of STAT3 signaling results in the reduced transcriptional activity of c-Myc and the diminished expression of the anti-apoptotic protein Bcl-2, thereby promoting apoptotic susceptibility in TNBC cells. Through this mechanism, SIRT1 operates as an essential molecular intermediary that translates CSE inhibition into enhanced responsiveness to chemotherapeutic agents [107].

In a recent study, Akbaribazm et al. showed that SIRT1 acts as an anti-metastatic and potentially tumor-suppressive regulator in 4T1 cell line xenografts. Cotreatment with *Trifolium pratense* L. extract and DOX upregulated SIRT1 gene expression, which coincided with a reduced metastatic tumor burden in the lung and brain, decreased IL-6 and IL-8, and the suppression of markers associated with metastatic dissemination (MMP-2, CK5/6, and GATA-3-positive cells) [108].

Nezamdoost et al. showed that SIRT1 modulates the cellular response to combined metabolic and phytochemical interventions. In 4T1 cells, concurrent high-intensity interval training and aqueous saffron extract significantly increased SIRT1 mRNA expression, suggesting a potential synergistic effect of exercise-induced stress and saffron bioactives in modulating epigenetic pathways within the tumor microenvironment. In addition, this SIRT1 upregulation was not accompanied by changes in p53 expression, indicating that the antitumor effects may proceed through p53-independent mechanisms. Given the role of SIRT1 in cellular stress responses and apoptosis regulation, its increased expression may reflect the activation of alternative tumor-suppressive processes to support SIRT1 as a possible mediator of non-pharmacological strategies aimed at modifying tumor behavior [109].

Zhou et al. demonstrates that SIRT1 acts as a suppressor of CSC-related transcriptional activity, and its degradation represents a pivotal step through which oxidized ATM sustains stemness in TNBC. In this study, ATM activates TRIM21, through phosphorylation, enabling it to bind and ubiquitinate SIRT1, thereby promoting its proteasomal degradation. As SIRT1 levels decline, its deacetylase activity toward histone H4 is reduced, resulting in heightened H4 acetylation and the subsequent activation of CSC-associated gene expression programs. This shift in chromatin state facilitates the preservation of BC stemness. Clinical observations support this mechanism, showing that tumors with high ATM expression correspondingly display low SIRT1 and high p-TRIM21 abundance alongside the increased expression of malignancy markers such as CD44 [110].

Table 4 summarizes the factors that regulate or are regulated by SIRT1 in driving its tumor suppressor role in TNBC.

In conclusion, SIRT1 plays a complex and context-dependent role in TNBC by acting as an oncogene or tumor suppressor and regulating key processes like lysosomal function, redox balance, and apoptosis, influencing tumor progression, angiogenesis, metastasis, chemotherapy resistance, stemness and EMT. Its involvement in resistance to treatment makes it a potential therapeutic target, but further research is needed to clarify its dual role and optimize its use in treatment strategies.

## 5. Modulators of SIRT1 in Breast Cancer

Modulating SIRT1 activity, through either inhibition or activation, has emerged as a promising therapeutic approach for BC. Given the aggressive characteristics and the limited availability of targeted treatments for TNBC, developing compounds that specifically target SIRT1 could offer a novel strategy to address the challenges associated with this highly metastatic and treatment-resistant cancer [111]. However, recently discovered natural compounds such as polyphenols including resveratrol (RES), fisetin, quercetin, and curcumin, as well as synthetic compounds, have been shown to enhance SIRT1 activity [112,113].

It is well known that RES, a phenolic compound, is a strong activator of SIRT1, with some of its biological effects being driven by the activation of SIRT1. Kala et al. demonstrated the effects of RES, in combination with pterostilbene, on TNBC cells. The combination was shown to downregulate SIRT1, leading to the reduced expression of γ-H2AX, a marker for DNA damage, and telomerase activity, both critical for tumor cell survival. This downregulation significantly inhibited the growth and migration of TNBC cells such as HCC1806 and MDA-MB-157 while inducing cell cycle arrest and apoptosis. In addition, this combination treatment also led to the downregulation of DNMTs, enzymes that are key players in the epigenetic regulation of gene expression, often altered in BC cells. No significant effects were observed in normal breast epithelial cells such as MCF-10A, indicating that the treatment selectively targets cancer cells without affecting normal cells [114].

Moreover, Fukui et al. investigate the role of SIRT1 and RES in mediating resistance to PTX in TNBC. The findings reveal that SIRT1 is essential for HER3 expression in response to RES. Silencing SIRT1 leads to a significant reduction in HER3 levels, preventing RES from rescuing cells from PTX-induced cytotoxicity. Interestingly, in the absence of SIRT1, RES itself induces higher cell death, highlighting a potential toxicity of RES when SIRT1 is depleted. In addition, RES activates SIRT1, which in turn regulates FOXO1 and FOXO3, whereas the knockdown of FOXO1 abolishes the protective effect of RES against PTX. The SIRT1/FOXO1 signaling pathway is crucial for the RES-mediated induction of HER3, which in turn facilitates resistance to PTX in BC cells, suggesting that the simultaneous use of RES and PTX is ineffective for treating BC expressing HER3 [115]. RES has also shown antitumor effects and can help overcome DOX resistance in MCF-7/ADR cells by modulating pathways involved in EMT. Indeed, when combined with DOX, RES significantly reduces cell viability, inhibits migration, and promotes apoptosis in resistant cells. RES modulates the interaction between SIRT1 and β-catenin, enhancing DOX sensitivity and reducing EMT features [116].

In the context of SIRT1 activators, Zhang et al. showed that quercetin 3,5,7,3′,4′-pentamethyl ether from *Kaempferia parviflora* (KPMF-8) emerges as a natural activator of SIRT1 more than RES, binding directly to its N-terminal domain and enhancing the enzyme affinity for its substrate acetylated-p53, thus contributing to the survival of BC cells and enabling resistance to therapy. Additionally, experiments on MCF-7 cells indicate that KPMF-8 is cell-permeable, suggesting that it can act directly within tumor cells [117].

In a study, SIRT1 emerges as a regulator of osteopontin (OPN) expression in proliferation, migration, and invasion processes in BC cells. RES reduces secreted OPN levels and clonogenic capacity in MDA-MB-435 and MDA-MB-231 cells, supporting its antitumor activity mediated by epigenetic mechanisms. Similarly, genistein also increases SIRT1 expression and markedly decreases secreted OPN levels, without direct transcriptional repression, as indicated by the increased OPN promoter activity observed in luciferase assays. Both genistein and RES modulate OPN expression through the involvement of the MAPK signaling pathway, whereas the NF-κB and PKB/AKT pathways do not appear to play a major role in this cellular context [118].

Pro-senescence therapy is a newly suggested approach for cancer treatment. Recently, Bian et al. proved that the activation of SIRT1 through RES treatment promotes cellular senescence in breast and lung cancer cells. This activation upregulates the p38MAPK pathway, triggering stress responses that result in irreversible cell growth arrest. SIRT1 also reduces nitric oxide levels, enhancing the expression of DLC1, a growth-suppressing protein. Additionally, RES-driven SIRT1 activation induces ER stress and mitochondrial dysfunction, leading to DNA damage and reinforcing the senescent phenotype [119].

In breast and lung cancer, SIRT1 regulates immune cell functions, particularly in neutrophils, which contribute to tumor metastasis. RES activates SIRT1 in neutrophils, inhibiting the formation of neutrophil extracellular traps (NETs). RES also increased CD8+ T cell infiltration in the lungs, suggesting enhanced immune response. Via SIRT1 activation, RES inhibits histone H3 citrullination, essential for NET formation; in contrast, neutrophils lacking SIRT1 showed increased NETs and enhanced metastasis in the lungs [120].

Nowadays, the resistance to chemo-radiotherapy (CRT) remains a central limitation in the treatment of TNBC. Based on a study, Fatehi et al. identified SIRT1 as a positive modulator of CRT responsiveness in TNBC. The pharmacological activation of SIRT1 using SRT1720 enhances the efficacy of CRT, particularly in the presence of IL-6, a tumor-associated cytokine that influences therapy resistance. SIRT1 activation not only increases the overall CRT efficacy but also sensitizes CSCs to radiation, especially when combined with the PI3K/AKT/mTOR inhibitor NVP-BEZ235. These findings suggest that SIRT1 activity contributes to overcoming therapeutic resistance and improves the in vitro therapeutic outcome of combined CRT regimens [121].

It was hypothesized that IL-6 exerts its effects through SIRT1, activating key cellular pathways such as PI3K. The findings shown by Masoumi et al. indicate that activating SIRT1 with SRT1720 enhanced the sensitivity of BC cells to radiotherapy, but also resulted in an increase in CSC populations, which are often linked to poor treatment outcomes. Interestingly, IL-6 pretreatment reduced CSC populations; however, when SIRT1 was inhibited using EX-527, the beneficial effects of IL-6 pretreatment were diminished, highlighting that SIRT1 inhibition interferes with IL-6 ability to regulate CSCs and cell viability. Additionally, the combination of NVP-BEZ235, a dual inhibitor of PI3K/mTOR, with SRT1720 significantly reduced cell viability and enhanced the effectiveness of radiotherapy, though it also led to an increase in CSCs. Overall, the study underscores that SIRT1 has a complex role in BC treatment [122].

On the other hand, as known, sirtinol is a potent SIRT1 inhibitor. A study conducted by Satam et al. examines the effects of sirtinol on EMT, metastasis, and the immune microenvironment in 4T1 BC cells. The research demonstrates that sirtinol is cytotoxic to BC cells and significantly impairs their ability to metastasize. This outcome is linked to its ability to inhibit EMT, as shown by a reduction in vimentin expression and decreased cell mobility. The study also explores how sirtinol influences the immune microenvironment in tumor-bearing mice. Indeed, treated mice showed notable changes in immune cell populations, including an increase in CD11b+ cells and a decrease in T cells, accompanied by the elevated levels of IFN-γ. Sirtinol has shown a dual action in both limiting tumor spread and modulating immune responses [123].

In the EMT context, El-Ashmawy et al. showed that, in both in vivo and in vitro models, treatment with berberine and sirtinol reduced EMT by downregulating SIRT1 expression associated with an increase in E-cadherin and a decrease in vimentin levels. The inhibition of SIRT1 led to suppression of the AKT signaling pathway, as evidenced by the reduced phosphorylation of AKT and lower levels of VEGFR2, both of which are integral to cancer cell survival, migration, and angiogenesis. These findings further indicate that SIRT1 contributes to the regulation of EMT and metastasis in BC through the modulation of the AKT/VEGFR2 axis [124].

SIRT1 has been implicated in drug resistance and operates in close association with the AKT signaling pathway. In DOX-resistant BC cells, i.e., MCF-7/ADR, SIRT1 is active within the cell nucleus, and the phosphorylation of AKT further enhances its activity, hindering the effectiveness of chemotherapy treatment. Nicotinamide (NAM), a known inhibitor of SIRT1 when administered in combination with DOX, induces the translocation of SIRT1 from the nucleus to the cytoplasm, dissociating it from the AKT pathway. This disruption blocks the action of SIRT1, leading to a reduction in AKT activation and lowering the expression of both SIRT1 and phosphorylated AKT. As a result, SIRT1 inhibition leads to decreased cell growth, suppression of tumor cell migration, and promotion of apoptosis in DOX-resistant cells [125].

Prasad et al. present a new approach to synthesizing 2-amino-1,3,4-thiadiazole derivatives, compounds with notable anticancer properties. The synthesized compounds were tested for their cytotoxic effects on MDA-MB-231 and MCF-7 metastatic BC cell lines, showing promising results in inhibiting cell growth. Among them, compound **3i** stood out as a strong inhibitor of SIRT1, sensitizing cancer cells to chemotherapy and improving treatment effectiveness [126].

In a recent investigation, the selective SIRT1 inhibitor Selisistat (EX-527) was employed to assess its effects on MDA-MB-231 cells. The results demonstrated that EX-527 significantly inhibited cell proliferation and induced apoptosis, primarily by reducing SIRT1 expression. Additionally, the migration and invasion capacities of these cancer cells were notably diminished. Moreover, the downregulation of SIRT1 was linked to the increased expression of p53, while the expression of POLD1, a gene essential for DNA replication and genomic stability, was also reduced, thereby influencing critical processes involved in cancer cell survival and cancer progression [127].

Notably, Wawruszak et al. demonstrated that the combination of EX-527 and PAX in a 1:1 ratio exhibited a synergistic effect significantly impairing luminal and TNBC cell growth. In addition, an in silico analysis revealed a potential protein–protein interaction pathway that connects the molecular targets of both compounds, which together contribute to a stronger therapeutic response. This pathway involves SIRT1, AKT, S1PR1, GNAI1/GNAO1, and tubulin. In xenograft models using zebrafish, the combination treatment resulted in a more substantial reduction in tumor growth compared to either drug alone [128].

SIRT1 inhibitors, particularly those derived from isoflavone and benzoylbenzofuran analogs, have shown promising potential for the treatment of TNBC. Selepe et al. tested isoflavone derivatives and found that these compounds exhibit potent antiproliferative effects on MDA-MB-231 cells, significantly reducing SIRT1 activity to levels comparable to the reference compound suramin. The potency of these compounds (particularly 28, 32, 38, 39, and 40) was also studied in silico using the crystal structure of SIRT1; notably, the 6-methoxy-4′,6′-dimethylisoflavone-2′,5′-quinone (compound 39) demonstrated strong SIRT1 inhibitory activity, and its optimal activity by its interaction in the active site of SIRT1 is comparable to suramin, suggesting that these compounds may enhance the efficacy of conventional therapies, particularly against drug-resistant cancer cells [129].

SIRT1 acts as a regulator of metastatic and immunosuppressive pathways in TNBC. In vivo treatment with anthocyanin-rich dark sweet cherry extracts (ACN) markedly reduced SIRT1 expression, indicating that ACN can modulate SIRT1-dependent signaling that supports tumor spread and immune evasion. Concurrent decreases in STAT3, Snail1, and mTOR suggest that ACN interferes with SIRT1-mediated deacetylation processes that drive EMT, metastatic progression, and TGFβ1-associated immunosuppressive activity. In addition, ACN disrupted the usual associations between SIRT1 and other markers, including CD44, Rgcc32, mTOR, and Snail1, highlighting its potential to destabilize SIRT1-centered regulatory networks that contribute to TNBC aggressiveness [130].

Highlighting the potential of SIRT1 inhibition in BC, MHY2256, another SIRT1 inhibitor, was found to significantly decrease cell viability in both p53-wild-type (MCF-7) and p53-null (SKOV-3) cell lines. This inhibitor demonstrated the potent suppression of SIRT1 enzymatic activity, leading to a notable reduction in the expression of SIRT1, SIRT2, and SIRT3. Furthermore, treatment with MHY2256 resulted in the increased acetylation of p53, enhancing its pro-apoptotic activity and inducing cell cycle arrest. In addition, MHY2256 promoted autophagic cell death [131].

A study carried out by Tenhunen et al. explored the impact of Bromodomain and Extra-Terminal motif (BET) inhibitors on SIRT1 in various cell lines. The compound (+)-JQ1 was found to lower SIRT1 levels in MDA-MB-231 cells, which contrasts with prior research where similar inhibitors led to an increase in SIRT1 in different cell lines. On the other hand, I-BET151 caused an increase in SIRT1 in both MDA-MB-231 and MCF-7 cells, while Pfi-1 did not alter SIRT1 levels, despite being a potent BET inhibitor. Additionally, the study observed variations in acetylated p53 levels in response to changes in SIRT1. In MDA-MB-231 cells, (+)-JQ1 reduced SIRT1 and simultaneously increased acetylated p53 levels, while I-BET151 raised SIRT1 without significantly affecting acetylated p53. This suggests that other mechanisms beyond SIRT1 might play a role in regulating p53 acetylation and that the effects of BET inhibitors on SIRT1 are complex and influenced by both the cell type and the specific inhibitor used [132].

A new strategy was developed using activity-based probes (ABPs) to directly assess SIRT1 activity in vitro and within cellular extracts. These ABPs were designed with four specific components to analyze the SIRT1 activity. The study further explored how modulating SIRT1 activity, through competition with acyl-lysine peptides, pharmacological inhibition, and post-translational modifications resulted in a loss of ABP labeling, indicating a decrease in SIRT1 activity within the subcellular environment of MCF-7 cells. The development of this more sensitive, SIRT1-specific ABP offers a powerful tool for the creation of chemical probes targeting this protein [133].

A recent study explored a novel pH-sensitive nanocomposite, FAHA-Amygdalin@Fe_2_O_3_ (AF), designed for targeted BC therapy. This nanocomposite releases its therapeutic agents in response to the acidic pH typical of tumor environments, enhancing the efficacy of treatment. Additionally, AF was tested in combination with γ-radiation to assess potential synergies. The study showed that AF nanoparticles exhibited sustained, pH-responsive release and selectively targeted BC cells. When treated with AF alone or with radiotherapy, BC cells experienced significant cell cycle arrest, increased apoptosis, and reduced tumorigenic potential. These effects were attributed to the inhibition of key oncogenic SIRT1, the restoration of p53 activity, and the disruption of YAP/TAZ, TGF-β/SMAD3, and HIF-1α/VEGF signaling [134].

Finally, to investigate the therapeutic potential of SIRT1 inhibition, a lentiviral vector expressing shRNA was also employed to selectively downregulate SIRT1 in BC cells. The findings revealed that silencing SIRT1 resulted in a notable reduction in cell proliferation, invasion, and migration in both the MDA-MB-231 and SK-BR-3 cell lines and enhanced apoptosis. At the molecular level, the depletion of SIRT1 led to the upregulation of tumor-suppressive markers such as α-catenin, PTEN, and E-cadherin, while simultaneously decreasing the expression of markers linked to EMT and tumor aggressiveness, including N-cadherin, β-catenin, and vimentin [135].

**Figure 2:**
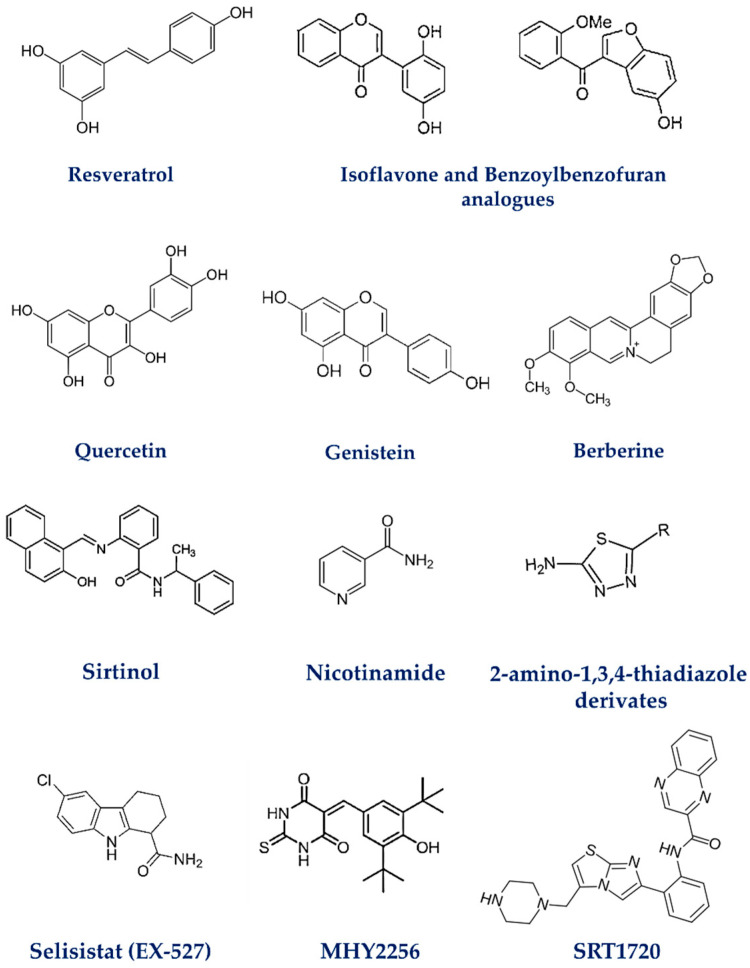
SIRT1 modulators.

In summary, the modulation and the targeting of SIRT1 using polyphenolic and synthetic compounds (Figure 2), BET inhibitors, ABPs, nanocomposites, and shRNA lentiviral vectors emerge as promising therapeutic approaches for BC including TNBC.

## 6. SIRT1 and miRNAs Network

The modulation of SIRT1, via miRNAs (miRs) and vice versa, plays a critical role in BC influencing tumor behavior. SIRT1 is controlled by various miRNAs that impact its expression altering the BC cell response to stress, inflammation, and hormonal signaling, all of which are key factors in the pathogenesis of BC. MiRNAs regulate cancer progression by targeting SIRT1, which can function as both oncogenes and tumor suppressors, depending on the specific pathway in each tumor type [136].

Current studies have underscored the critical role of lncRNAs in BC. In particular, lncRNA-PRLB has been found to be upregulated in BC and positively correlated with poor prognosis, and this regulation involves the inhibition of SIRT1. The study by Liang et al. identified miR-4766-5p as an inhibitory target of lncRNA-PRLB. In this context, SIRT1 was found to be negatively regulated by miR-4766-5p, as evidenced by its reduced expression following both lncRNA-PRLB knockdown and miR-4766-5p overexpression. Interestingly, the effects of lncRNA-PRLB overexpression, including the increased SIRT1 expression, were partially reversed by the ectopic expression of miR-4766-5p, suggesting that the modulation of SIRT1 plays a pivotal role in the progression of BC [137].

In this context, a recent study explored the miR-200a/lncRNA H-19 axis and its interaction with the IL-6/SIRT1 pathway, focusing on metastasis-related gene expression changes. Results showed the increased levels of lncRNA H-19, miR-200a, and IL-6 in BC patients, while SIRT1 was downregulated, and metastatic BC patients had higher levels of these biomarkers compared to non-metastatic BC patients. A negative correlation was found between lncRNA H-19 and miR-200a expression and SIRT1 levels, suggesting that elevated H-19 and miR-200a reduce SIRT1 expression, promoting tumor growth and metastasis. Additionally, both biomarkers were positively correlated with IL-6. The IL-6/SIRT1 axis, together with lncRNA H-19 and miR-200a, offers promising potential for diagnostic and therapeutic strategies, with the possibility to modulate SIRT1 and inhibit tumor progression [138].

Moreover, another study investigated the potential use of lncRNA PVT1/miR-146a/SIRT1 axis as a promising biomarker pathway for the early detection and prognostic evaluation of BC. The findings revealed that both lncPVT1 and SIRT1 were elevated in BC patients, whereas miR-146a showed reduced levels. In patients with metastatic BC, the levels of lncPVT1 and SIRT1 were notably higher compared to those with non-metastatic BC, suggesting a link between SIRT1 expression and the severity of the tumor. Additionally, lncPVT1 was found to have a positive correlation with SIRT1 and a negative correlation with miR-146a, implying that lncPVT1 may enhance SIRT1 expression while inhibiting miR-146a [139].

Yarahmadi et al. investigated how miR-211-5p affects the SIRT1/p53 signaling pathway and how it influences the viability and apoptosis of BC cells. To manipulate miR-211-5p levels, cells were transfected with either a miR-211-5p mimic or inhibitor. The findings revealed that miR-211-5p downregulated SIRT1 expression by directly binding to the 3′-UTR of its mRNA, leading to decreased SIRT1 gene and protein levels. This reduction in SIRT1 expression also resulted in a decrease in its enzymatic activity and in the acetylation status of p53, which consequently impaired cell viability and promoted apoptosis. Furthermore, inhibiting miR-211-5p using antisense oligonucleotides reversed these effects, restoring SIRT1 expression and reducing apoptosis, thus confirming that miR-211-5p plays a negative role in regulating BC cell survival [140].

Another research focuses on understanding the role of SIRT1 in the development of TAM resistance in BC cells and how its expression is regulated by the OAS1/miR-22-3p pathway. In TAM-resistant cell lines MCF-7TR and T47DTR, SIRT1 expression is found to be elevated. Additionally, OAS1 was shown to be highly expressed in TAM-resistant cells and acts as a miR-22-3p sponge, thereby reducing the expression of miR-22-3p and increasing SIRT1 levels. The study demonstrated that corylin upregulated miR-22-3p, leading to the suppression of OAS1. This suppression then resulted in decreased SIRT1 expression, linking miR-22-3p and OAS1 to the regulation of SIRT1 and revealing that the OAS1/miR-22-3p/SIRT1 axis is a critical pathway in TAM resistance in BC [141].

Ilisso et al. investigated the modulation of miRNA expression profiles induced by AdoMet in MCF-7 cells and found that miR-34a and miR-34c modulate the expression of SIRT1 and HDAC1. Overexpression of miR-34a and miR-34c resulted in a marked decrease in the levels of both SIRT1 and HDAC1, with this effect being further enhanced when combined with AdoMet. This reduction in SIRT1 and HDAC1 was associated with a significant increase in the acetylation of p53, suggesting that miR-34a and miR-34c by inhibiting SIRT1 facilitate the restoration of p53 tumor-suppressing functions [142].

In TNBC, a major hurdle in treatment is the development of resistance to DOX when a crucial factor in this resistance is the regulation of SIRT1. The microRNA-449 family (comprising microRNA-449a, -449b-5p, and -449c-5p) is significantly downregulated in TNBC, and this alteration has been linked to chemotherapy resistance. The mechanism behind this involves a negative feedback loop where the microRNA-449 family suppresses SIRT1, which in turn interacts with HDAC1 to regulate cellular responses to chemotherapy. This negative regulation of SIRT1 contributes to cellular survival and helps the BC cells evade the effects of the drug. Additionally, this loop affects mechanisms like the ABCG2 efflux pump, which is known to play a role in resistance to DOX [143].

A recent investigation also explored the potential impact of the GAS5 rs145204276 ins/del variant on the expression levels of SIRT-1, TGF-β, and miR-182, with the goal of evaluating their diagnostic relevance in BC. The relationship between miR-182 and SIRT1 is of particular interest, as emerging research suggests that SIRT1 may influence miR-182 expression in tumor cells. Specifically, SIRT1 could directly promote the upregulation of miR-182, which might then affect other key signaling pathways, such as the TGF-β pathway. This interaction between SIRT1, miR-182, and TGF-β seems essential for tumor dissemination and may contribute to BC aggressiveness [144].

The involvement of SIRT1 in BC was also analyzed in relation to its regulation by miR-590-3p. The study conducted by Abdolvahabi et al. observed that miR-590-3p expression is notably downregulated in BC cells and was coupled with an increase in SIRT1 expression. The research demonstrated that miR-590-3p directly targets the mRNA of SIRT1, leading to a significant decrease in both the protein levels and activity of SIRT1. This inhibition of SIRT1 by miR-590-3p subsequently affected critical cellular functions related to survival and apoptosis. Specifically, the upregulation of miR-590-3p resulted in the elevated acetylation of p53, leading to an increase in the expression of pro-apoptotic factors such as BAX and p21, which in turn reduced cell survival and triggered apoptosis in BC cells [145].

In a study by Jia et al., miR-301 was shown to be significantly overexpressed in BC, driving progression and metastasis. Specifically, the study suggests that high levels of miR-301 suppress CPEB1 expression, which in turn upregulates the SIRT1/SOX2 pathway, accelerating BC progression. Furthermore, SIRT1 regulates SOX2 expression, which was also found to be upregulated in cells with CPEB1 knockdown. Additionally, the mechanism by which miR-301-mediated CPEB1/SIRT1/SOX2 signaling exerts its inhibitory effect was confirmed in nude mice with xenografted BC [146].

A recent study explored the involvement of miR-29a in BC associated with T2DM, highlighting that miR-29a is significantly overexpressed in these patients and linked to a worse prognosis. The interaction between miR-29a and SIRT1 appears to facilitate tumor growth and metastasis, which are reduced with miR-29a knockdown. The findings were confirmed in vitro and in a xenograft mouse model [147].

Finally, miR-155-5p originating from myeloid-derived suppressor cells plays a significant role in regulating the invasiveness and migration of BC cells. Differential gene expression analysis has identified SIRT1 as one of the primary targets of miR-155-5p, which directly downregulates its expression, promoting tumor invasiveness and EMT, as confirmed by luciferase reporter assays. Furthermore, the overexpression of SIRT1 reversed these effects, suggesting that SIRT1 functions as a tumor suppressor, and, in PIK3CA-mutated animal models, the inhibition of miR-155-5p significantly decreased tumor growth, concomitant with increased SIRT1 levels and reduced EMT markers [148].

**Figure 3:**
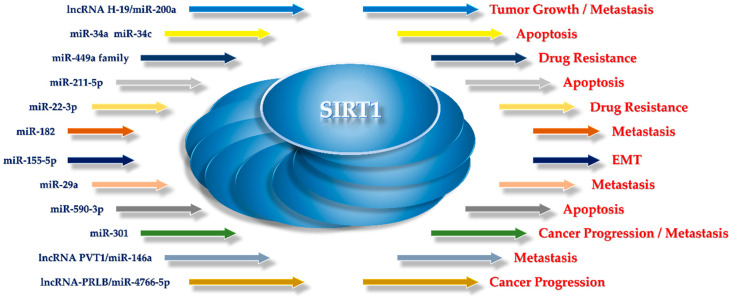
MicroRNAs involved in the regulation of SIRT1 in processes related to BC.

Overall, the findings from the past ten years of research presented in this review support the hypothesis that the regulation of SIRT1 occurs through miRNA modulation (Figure 3), and vice versa, and it could represent a promising strategy for the development of novel targeted therapeutic approaches for BC treatment.

## 7. Conclusions

SIRT1 stands out as a pivotal regulator in BC biology, influencing a wide range of processes and exerting a dual role as both an oncogene and a tumor suppressor, depending on the cellular and molecular contexts. Particularly, SIRT1 plays a crucial role in maintaining genomic stability, regulating cell cycle checkpoints, and facilitating DNA repair, acting as an essential mechanism for safeguarding against tumor progression; however, its activity may be associated with conflicting outcomes. In TNBC, elevated SIRT1 expression seems to be correlated with poor prognosis, EMT, and mechanisms of chemotherapy resistance through the involvement of CSCs. The finding that SIRT1 can be modulated not only through pharmacological approaches but also via natural extracts paves the way for alternative or complementary therapeutic strategies in the treatment of BC. Furthermore, the modulation of SIRT1 is influenced by a complex network of miRNAs, which warrants further investigation. Additional studies are required to better define SIRT1 role and optimize its clinical applications as a therapeutic target in BC subtypes. Although preclinical research has expanded substantially with recent advances in SIRT1 modulators, clinical translation remains in its early stages.

## Figures and Tables

**Table 1:** Main BC molecular subtypes.

BREAST CANCER MOLECULAR SUBTYPES			
Luminal A	Luminal B	HER2−Enriched	Basal-like (TNBC)
ER and/or PR+	ER and/or PR+	ER- PR-	ER- PR-
HER2−	HER2+ or HER2−	HER2+	HER2−
Low Ki-67 (<14%)	High Ki-67 (>14%)	High Ki-67 (>14%)	High Ki-67 (>14%) CK5/6 and/or EGFR+

ER: estrogen receptor; PR: progesterone receptor; HER2/neu: Human Epidermal Growth Factor Receptor 2; Ki-67: Marker of Proliferation Kiel 67; CK5/6: cytokeratin 5/6; EGFR: Epidermal Growth Factor Receptor; +: positive; -: negative.

**Table 2:** Classification, localization, substrates, and biological implications of SIRT1.

MAMMALIAN SIRT1				
Superfamily	Family	Class	Subclass	Localization
Deoxyhypusine synthase like NAD/FAD-binding domain	Sir2 regulator	Class III	I	Nucleus/Cytoplasm
**Histone Substrate**	**Non-Histone Substrate**	**Biological Implication**		
H1K26ac, H3K9ac, H3K14ac, H3K56ac, H4K16ac	FOXO1, FOXO3, p53, PPARγ, PGC1α, E2F1, β-catenin, MyoD, MYC, PCAF, Ku70, NF-kB, HIF1α, HIF2α, Rb, LKB1	DNA repair, genome stability, autophagy, neurodegenerative and metabolic diseases, cellular stress, cancer		

**Table 3:** Oncogenic role of SIRT1 in TNBC. This table summarizes the molecular factors regulated by SIRT1 that promote tumor progression and influence clinical outcomes in TNBC.

ONCOGENIC SIRT1 IN TNBC		
Hallmark Process	SIRT1-Regulated Factors/Regulators	Functional Outcome
Survival, proliferation, and drug resistance	NRF2, GSH, AKT	Activation of antioxidant response and AKT signaling promotes redox balance, tumor cell proliferation, and resistance to apoptosis and chemotherapy
EMT	ZEB1, CBP, CtBP, Vimentin, Ubc9, E-Cadherin, Caveolin-1, ERα	Regulation of EMT transcription factors and markers promotes epithelial–mesenchymal plasticity and acquisition of a mesenchymal phenotype
Migration, invasion, and metastasis	AKT, Ubc9, DVL	Activation of AKT and modulation of Wnt signaling enhance cell motility, invasiveness, and metastatic dissemination
Angiogenesis	VEGF	Upregulation of VEGF promotes tumor vascularization and supports tumor growth
CSCs maintenance and self-renewal	OCT4, SOX2, NANOG, Visfatin, ALDH1A1, c-Myc, SIRT4, p53	Regulation of stemness-associated factors enhances CSCs phenotype, sphere formation, and aggressiveness

**Table 4:** Tumor-suppressive role of SIRT1 in TNBC. This table summarizes molecular factors regulated by SIRT1 that counteract tumor progression and may contribute to improved clinical outcomes in TNBC.

TUMOR SUPPRESSOR SIRT1 IN TNBC		
Hallmark Process	SIRT1-Regulated Factors/Regulators	Functional Outcome
Lysosomal function and exosome regulation	ATP6V1A, IGF2BP2	Maintains lysosomal acidification and proper exosome cargo, preventing ECM remodeling and invasion
EMT inhibition	NF-κB, p66Shc, E-Cadherin, Vimentin, Snail1/2	Suppresses EMT, increases epithelial markers, decreases mesenchymal markers, reducing migration and invasiveness
Apoptosis and drug resistance	p53, c-Myc, STAT3, Bcl-2	Enhances apoptotic susceptibility and chemosensitivity, downregulating oncogenic and anti-apoptotic signaling
Metabolic stress adaptation and motility	AMPK, β1-integrin	Integrates energetic stress signals to promote cytoprotection while limiting adhesion and migration
CSCs inhibition and stemness suppression	ATM, TRIM21, H4 acetylation	Prevents activation of CSCs transcriptional programs, reducing stem-like properties and tumor aggressiveness

## Data Availability

No new data were created or analyzed in this study.
